# Precision immunotherapy with CAR-T cells in pediatric B-cell acute lymphoblastic leukemia: advances and unanswered challenges

**DOI:** 10.3389/fonc.2025.1691189

**Published:** 2026-01-14

**Authors:** Fu Li, Libo Zheng

**Affiliations:** Department of Hematology and Oncology, Children's Hospital Affiliated to Shandong University, Jinan, Shandong, China

**Keywords:** CAR-T therapy, cytokine release syndrome, immune effector cell-associated neurotoxicity syndrome, pediatric B-ALL, tisagenlecleucel

## Abstract

Chimeric antigen receptor (CAR) T-cell therapy has emerged as a groundbreaking treatment for pediatric B-cell acute lymphoblastic leukemia (B-ALL), especially for patients with relapsed or refractory disease. CD19-targeted CAR T cells, such as tisagenlecleucel, have demonstrated high rates of complete remission and long-lasting responses in clinical trials. However, challenges such as cytokine release syndrome (CRS), immune effector cell-associated neurotoxicity syndrome (ICANS), antigen escape, and T-cell exhaustion hinder its broader clinical application. Recent advances aim to overcome these obstacles by using multi-targeted CAR-T constructs (e.g., CD19/CD22), creating armored CAR-T cells with enhanced cytokine signaling, and developing optimized combination therapies. Next-generation approaches, including universal CAR-T cells and microenvironment-responsive designs, show promise in improving efficacy and safety. Despite these innovations, further research is needed to refine manufacturing processes, reduce costs, and improve long-term outcomes. This review emphasizes the transformative potential of CAR-T therapy for pediatric B-ALL and discusses critical challenges and future directions in the field.

## 1 Introduction

B-ALL is the most prevalent childhood malignancy, accounting for more than 80% of all pediatric leukemia cases (1). It arises from the malignant transformation of B-lineage precursor cells, leading to uncontrolled proliferation of immature lymphoblasts in the bone marrow, peripheral blood, and extramedullary sites, which in turn suppresses normal hematopoiesis (2, 3). Although advancements in intensive chemotherapy have elevated the cure rate in children to over 90%, around 10–15% of patients experience relapse or develop refractory(R/R) disease (4–6). These cases are linked to a poor long-term survival rate of around 30%-60%, and even worse with a second or later relapse, which poses a significant therapeutic challenge in pediatric oncology (7–10).

The standard treatment protocol for pediatric B-ALL typically involves risk-directed therapy and central nervous system (CNS) prophylaxis (11–13). For high-risk patients or R/R B-ALL, allogeneic hematopoietic stem cell transplantation (HSCT) was historically the main curative option, though its use is limited by toxicity, graft-versus-host disease (GVHD) and donor availability (14–16). Despite the remarkable success of conventional therapeutic modalities in the treatment of pediatric B-ALL, 15-20% of patients still fail treatment due to the presence of multiple resistance mechanisms (17). These mechanisms include inherent genetic abnormalities in leukemia-initiating cells (e.g., IKZF1 deletion (18), CRLF2 rearrangement (19), etc.), protective survival signals provided by the bone marrow microenvironment (20), clonal evolution that occurs under therapeutic pressure (e.g., TP53 mutation, loss of CD19 antigen) (21), and drug metabolic barriers (22). These limitations have prompted researchers to develop novel immunotherapies that can overcome traditional drug resistance. Among these novel immunotherapies, CAR-T cell therapy, which involves the genetic engineering of T cells to specifically recognize and remove tumor cells, offers a breakthrough treatment option for drug-resistant B-ALL. However, there are still major challenges to its clinical application, including antigen escape, drug resistance, and therapy-associated toxicities (23, 24).

## 2 Research progress of CAR-T cell therapy in pediatric B-ALL

CAR T-cell therapy represents a major advancement in the treatment of pediatric B-ALL, especially in relapsed or refractory cases with limited therapeutic options. Since the US Food and Drug Administration (FDA) approved tisagenlecleucel (Kymriah^®^) in 2017, CD19-directed CAR-T cells have demonstrated significant clinical efficacy (25). Recent developments include dual-targeted constructs (e.g., CD19/CD22), cytokine-armored CAR T cells, and other strategies (26, 27). This section outlines key advances in CAR-T cell research for pediatric B-ALL, emphasizing the rapid progress being made in this field and its therapeutic potential.

### 2.1 Mechanism of CAR-T cell therapy

**Figure 1:**
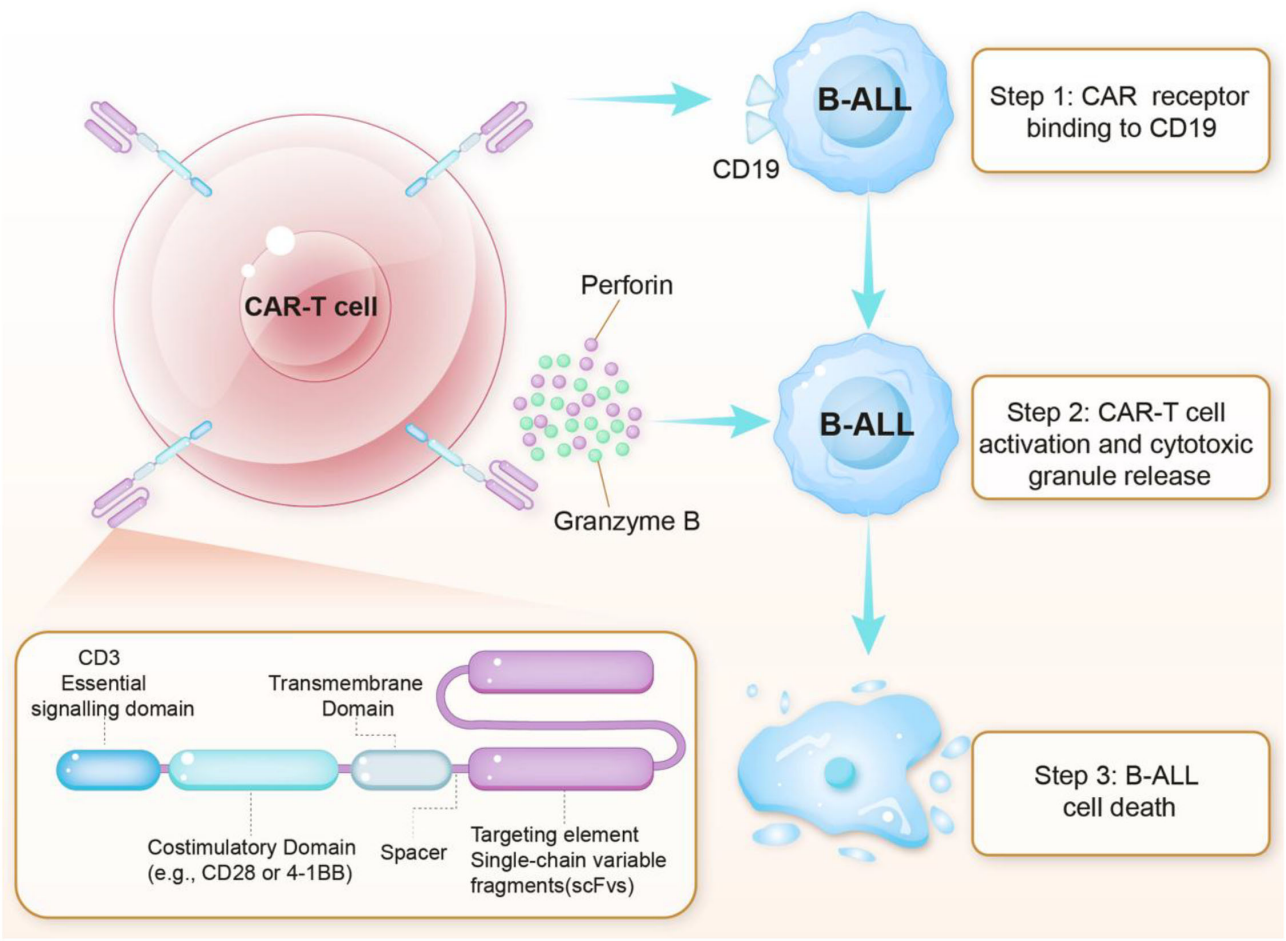
Molecular mechanism of CAR-T cell therapy for the B-ALL treatment. CAR-T cell structure: The engineered CAR is composed of an extracellular CD19-targeting scFv, a spacer region, a transmembrane domain, and intracellular signaling domains including the primary CD3ζ activation motif and a costimulatory domain (CD28 or 4-1BB) that enhances T-cell persistence and efficacy. Mechanism of action: Upon binding to CD19 on B-ALL cells, the CAR initiates T-cell activation through immunological synapse formation, thereby triggering the release of cytotoxic granules containing perforin and granzyme B. Perforin facilitates granzyme B entry into target cells, where it activates apoptotic pathways, while concurrent cytokine production (e.g., IFN-γ, TNF-α) and Fas/FasL interactions further amplify tumor cell death. This targeted mechanism selectively eliminates CD19+ malignant cells while sparing normal tissues, thereby demonstrating the precision of CAR-T immunotherapy. The choice of costimulatory domain (CD28 vs. 4-1BB) influences the kinetics and durability of the antitumor response.

CAR-T cell therapy is a transformative immunotherapeutic strategy that employs genetically modified T lymphocytes to eliminate malignant cells in pediatric B-ALL selectively (**Figure 1**). The recognition of surface antigens initiates the therapeutic process—primarily CD19—via synthetic chimeric antigen receptors (CARs), which are composed of an extracellular single-chain variable fragment (scFv), hinge region, transmembrane domain, and intracellular signaling modules (typically CD3ζ plus co-stimulatory domains such as CD28 or 4-1BB) (28–30). Preclinical and clinical studies have demonstrated that CAR-T cells with a 4-1BB co-stimulatory domain exhibit enhanced persistence and reduced terminal differentiation compared with CD28-based constructs, contributing to more durable antitumor responses in pediatric B-ALL patients (30, 31). Consequently, optimizing these intracellular domains has become a key focus in CAR-T design, aiming to balance robust antitumor activity with controlled cytokine release and improved safety profiles. Upon antigen engagement, CAR-T cells form immunological synapses with target cells and trigger activation cascades that drive proliferation and cytotoxic responses. These include perforin/granzyme-mediated lysis (32–34), Fas/FasL-induced apoptosis (35–37), and pro-inflammatory cytokine release (e.g., IFN-γ, TNF-α) (38, 39).

Unlike native T-cell responses, CAR-T cell recognition is independent of MHC presentation, enabling effective targeting even when leukemic cells downregulate MHC molecules, which is a common immune escape mechanism (40). In pediatric B-ALL, high CD19 expression and the proliferative capacity of pediatric T cells contribute to the robust clinical responses observed. However, potent immune activation can also lead to on-target effects, such as B-cell aplasia, as well as toxicities, including CRS (41).CAR-T cells exhibit a biphasic kinetic profile—characterized by rapid expansion, contraction, and long-term persistence—enabling sustained anti-leukemic activity. Functional CAR-T cells can remain detectable for months to years’ post-infusion in responding patients (42, 43). Notably, while maintaining long-term efficacy is the ideal goal, sustained clinical remission does not always depend on the prolonged persistence of therapeutic cells. This precise yet highly potent mechanism underpins both the unprecedented therapeutic outcomes and the unique challenges associated with CAR-T therapy in pediatric B-ALL and continues to guide next-generation CAR designs and clinical refinements.

### 2.2 Current status and major achievements of clinical trials

#### 2.2.1 CD19-targeting CAR-T cell

##### 2.2.1.1 Tisagenlecleucel

Tisa-cel, a landmark immunotherapy for treating pediatric B-ALL, is the result of over two decades of preclinical and clinical research. Early foundational research in the 1990s first demonstrated the potential of CARs to redirect T-cell specificity (44, 45). Key studies by Eshhar et al. established the basic scFv design, and subsequent optimization led to the development of second-generation CARs, which incorporated co-stimulatory domains (28, 46). The CAR structure consists of an anti-CD19 single-chain antibody (scFv derived from the FMC63 clone), a 4-1BB (CD137) co-stimulatory domain and a CD3ζ signal domain (30, 47). Comparative studies in xenograft models demonstrated that the 4-1BB (CD137) domain exhibited superior persistence to the CD28-based construct, a critical finding that directly informed the design of Tisa-cel (48). Clinical trials revealed that, compared to CAR-T with a CD28 co-stimulatory domain, Tisa-cel demonstrated fewer complications (49). This is associated with metabolic reprogramming, which promotes mitochondrial oxidative phosphorylation and reduces terminal differentiation. Additionally, improvements in lentiviral transduction efficiency (achieving 30–50% in clinical-grade production) and optimization of the T-cell expansion protocol ensure stable CAR expression (31, 50). This enables the reliable production of therapeutic doses and lays the foundation for long-term efficacy.

The selection of CD19 as the target antigen was guided by extensive biomarker analyses demonstrating its expression in over 95% of pediatric B-ALL blasts (51). Preclinical studies using the FMC63-derived antibody confirmed strong anti-leukemic efficacy, with off-target effects largely restricted to B-cell depletion (52). Pharmacokinetic evaluations in non-human primates further established a characteristic biphasic profile of CAR-T cells—initial expansion, subsequent contraction, and sustained persistence—later corroborated in human trials (50, 53, 54). These findings informed the design of a Phase I dose-escalation trial (NCT01626495), which identified an effective dosing range (0.2–5 × 10^6^ CAR-T cells/kg) and established critical safety benchmarks for clinical implementation (55, 56).

The international ELIANA trial (NCT02435849) enrolled 75 pediatric and young adult patients with B-ALL, a group characterized by limited treatment options and a high relapse risk. The intention-to-treat analysis showed a complete remission(CR) rate of 81%, with all responders achieving minimal residual disease (MRD) negativity by flow cytometry. Among those achieving remission, 59%(95%CI: 41-37) remained relapse-free, 50%(95%CI: 35-64) remained event-free survival(EFS) at 12 months, and the overall survival(OS) rate was 76% (95% CI, 63 to 86) (56). At the three-year follow-up, the CR rate was 82%, with an EFS rate of 44% (95% CI: 31–57) and an OS rate of 63% (95% CI: 51–73). Most adverse events occurred within the first two years. The estimated three-year relapse-free survival (RFS) was 52% (95% CI: 37–66) with censoring for subsequent therapies, and 48% (95% CI: 34–60) without censoring (57). The following table (**Table 1**) presents a summary of the available data regarding the real-life experience of Tisagenlecleucel. These clinical outcomes consistently highlight the advantage of the 4-1BB costimulatory domain in maintaining T-cell persistence, as evidenced by the durable responses observed. However, the observed variability in complete remission rates across different studies (ranging from 81% to 99.1%) suggests that patient-specific factors, including disease burden, prior treatment history, and tumor microenvironment characteristics, may significantly influence therapeutic outcomes.

**Table 1:** Clinical trials for tisagenlecleuce.

Reference	Infused	Age,year	CR	OS	EFS	MDR-negativity
Schultz LM et al. (58)	185	12(0-26)	85%	72% (1 year)	50% (1 year)	97%
Oporto Espuelas M et al. (59)	125	11(0.7-25.7)	92%	70% (2 years)	51.7% (2 year)	90.4%
Kato I et al. (60)	42	10(1-23)	92%	81.6% (1 year)	56.3% (1 year)	97.2%
Pasquini MC et al. (61)	255	13.2	99.1%	60.9% (1 year)	52.4% (1 year)	85.5%
Pasquini MC et al. (62)	144	13(2-26)	89%	89% (6 months)	66% (6 months)	100%

CR, complete remission; OS, overall survival; EFS, event-free survival; MRD, minimal residual disease.

##### 2.2.1.2 brexucabtagene autoleucel

Brexu-cel, a CD19-directed CAR-T cell therapy incorporating a CD28 costimulatory domain, is approved for treating R/R B-ALL. Like other CD28-based CAR-T constructs, brexu-cel is characterized by rapid T-cell activation, robust early expansion and potent cytotoxic effector function. This reflects the strong proximal signaling delivered through CD28-mediated costimulation (63).

A distinctive feature of brexu-cel manufacturing is the inclusion of a T-cell enrichment step, which reduces contaminating leukemic blasts and may enhance product potency, particularly in patients with high disease burden (64, 65). The clinical efficacy of brexu-cel has been demonstrated in the pivotal ZUMA-3 trial (65), which evaluated its use in adults with relapsed or refractory B-cell acute lymphoblastic leukaemia (ALL). The primary analysis showed that brexu-cel achieved a CR or CR with incomplete haematological recovery (CRi) rate of around 70%. The majority of patients who responded achieved minimal residual disease negativity. With extended follow-up, durable responses were observed in some patients (66). However, relapses remained common, highlighting the difficulty of achieving long-term disease control in this heavily pretreated population.

Consistent with the kinetic profile of CD28-based CAR-T therapies, brexu-cel treatment was associated with rapid CAR-T cell expansion and a relatively early peak in cytokine levels. Correspondingly, higher rates of inflammatory toxicities, including cytokine release syndrome and immune effector cell–associated neurotoxicity syndrome, were reported compared with 4-1BB–based products, although these adverse events were generally manageable with current supportive and immunosuppressive strategies (30, 67). CAR-T cell persistence following brexu-cel infusion was typically shorter than that observed with 4-1BB–containing CAR-T products, a feature that may influence relapse risk and inform post–CAR-T consolidation strategies, such as allogeneic hematopoietic stem cell transplantation, in selected high-risk patients.

##### 2.2.1.3 Axicabtagene ciloleucel

Axi-cel is a widely used CD19-directed CAR-T cell product whose design incorporates a CD19-specific single-chain variable fragment (scFv), a CD3ζ signaling domain, and a CD28 costimulatory module. This configuration was originally introduced by Kochenderfer et al. in 2009, who demonstrated its potent cytotoxic effects against CD19-positive B cell malignancies in preclinical studies (68). Axi-cel was subsequently translated into clinical application for relapsed or refractory large B-cell lymphoma (LBCL), and in the pivotal ZUMA-1 trial, it achieved an objective response rate (ORR) of 82% and a complete remission (CR) rate of 54% (69).

Although Axi-cel has demonstrated robust efficacy in adult LBCL, evidence for its use in pediatric or adolescent B-ALL remains limited. Current investigations in this age group are primarily focused on structurally related constructs such as KTE-X19, and formal clinical reports of Axi-cel in B-ALL are still lacking (70). However, studies of axi-cel have provided important mechanistic insights into CD28-mediated CAR-T cell signaling, kinetics, and effector differentiation. These findings have substantially contributed to the broader understanding of how costimulatory domains shape CAR-T cell biology. In this review, axi-cel is therefore discussed primarily as a mechanistic reference rather than as a disease-specific therapeutic comparator in ALL.

#### 2.2.2 CD22-targeting CAR-T cell

Although it is currently investigational and has not yet been approved by the FDA for B-ALL,CD22-targeting CAR T-cell therapy has emerged as a promising alternative for patients with relapsed/refractory B-cell malignancies, particularly those resistant to CD19-directed therapies. CD22, a sialic acid-binding immunoglobulin-like lectin (Siglec) expressed on mature B cells and most B-cell malignancies, plays a crucial role in B-cell receptor (BCR) signaling modulation (71). Unlike CD19, which is rapidly internalized upon CAR-T engagement, CD22 exhibits slower internalization kinetics, potentially enhancing CAR-T persistence and reducing antigen escape (72). The CAR structure typically incorporates an anti-CD22 scFv derived from monoclonal antibodies (e.g., m971 or epratuzumab), coupled with CD3ζ and co-stimulatory domains (4-1BB or CD28) to optimize T-cell activation and survival (73, 74).

CD22-targeted CAR-T cell therapy has been shown to be clinically valuable in treating B-ALL, particularly in patients who have not responded to CD19-targeted therapy. A study by Fry TJ et al. have shown that CD22 CAR-T achieves complete remission rates of 88% in adult B-ALL patients and 73% in pediatric patients (75). Notably, CD19/CD22 dual-target CAR-T (CAR1922T2) significantly reduces the risk of relapse due to antigen escape because it targets both antigenic sites simultaneously. This increases the complete remission rate to 99.1% and achieves a one-year event-free survival rate of 75.5% (76). A study showed that the sequential use of CD19 and CD22 CAR-T cells in B-ALL patients who relapsed after allogeneic HSCT resulted in 5-year overall and event-free survival rates of 75% and 50%, respectively (77). However, CD22 CAR-T therapy faces several challenges, including CD22 downregulation after treatment and B-cell depletion, leading to relapse of patients (78). In terms of safety, the incidence of severe cytokine release syndrome (grade ≥3) was lower with CD22 CAR-T than with CD19 CAR-T, but neurotoxicity remains a concern (79, 80). To improve therapeutic efficacy, investigators are exploring multiple optimization strategies. These include developing IL-15-secreting armored CAR-Ts (81) to enhance cellular persistence and creating universal CD22 CAR-Ts based on CRISPR gene editing technology (26). These advances provide new therapeutic options for patients with B-ALL who have failed CD19 therapy and help overcome the limitations of current treatments. Future research will focus on improving treatment response durability and developing more effective combination therapies. It is important to note that the comparisons of efficacy and toxicity between CD19 and CD22 CAR-T cells are based on cross-trial analyses rather than direct head-to-head randomized trials. Differences in patient populations, trial designs, and endpoint definitions may influence the observed outcomes.

CAR-T therapy for B-ALL has evolved through distinct targeting approaches, each with characteristic efficacy and safety profiles. CD19-directed therapy has been shown to yield higher complete response rates (81-99% vs. 73-88% for CD22) but greater toxicity, including higher rates of severe CRS (22-46% vs. 10-28%) and ICANS (13-31% vs. 5-18%) (56, 75). The CD19/CD22 dual-targeting strategy (76, 82) has been shown to combine the advantages of these two approaches while mitigating the limitations inherent to each. This strategy has been found to achieve superior efficacy, with a CR rate of 99%, while also maintaining intermediate toxicity, with grades of CRS ≥3 of 15-35% and ICANS of 8-25%. This approach has been demonstrated to reduce antigen escape (<5% vs. 30-50% for single-target) through complementary mechanisms: CD22’s protracted internalization (t1/2>24h vs. CD19’s ~4h) augments persistence while sustaining B-cell depletion (75, 83, 84). However, a number of challenges were also identified, including suboptimal CD22 targeting and accelerated T cell exhaustion (85). These advances underscore the importance of balancing enhanced anti-leukemic activity with manageable toxicity in next-generation CAR-T designs, while also highlighting the need for long-term follow-up of dual-target strategies.

For a detailed overview and direct comparison of these clinical studies, please refer to **Supplementary Table S1**, which lists the principal trials, targets, product types, research centers, and main efficacy and safety results described in this review.

## 3 Current challenges in CAR-T cell therapy

**Figure 2:**
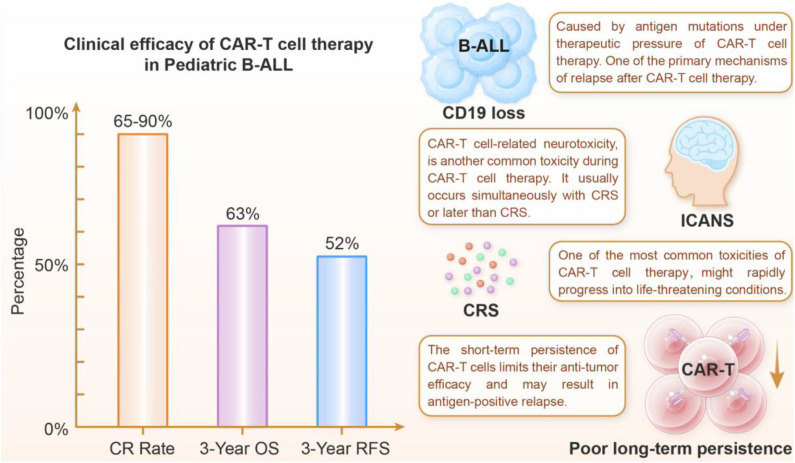
The clinical efficacy and key challenges of CAR-T cell therapy for pediatric B-ALL. CR, complete remission; OS, overall survival; RFS, relapse-free survival; ICANS, immune effector cell-associated neurotoxicity syndrome; CRS, cytokine release syndrome; ASTCT, American Society for Transplantation and Cellular Therapy. Clinical efficacy: The left section shows treatment outcomes, showing high CR rates (81-99.1%) (56, 61), with 3-year OS at 63% and RFS at 52% (57). Major limitations: (1) CD19 antigen loss has been observed in 30-50% of relapses, primarily through alternative splicing (Δex2) or lineage switching, resulting in antigen-positive relapse; (2) ICANS, evaluated according to ASTCT criteria, affects 13-31% of patients (grade ≥3) and manifests as neurological symptoms ranging from aphasia to cerebral edema, driven by cytokine-mediated blood-brain barrier disruption; (3) CRS, a systemic inflammatory response characterized by fever, hypotension, and organ dysfunction, occurs in 22-46% of cases (grade ≥3) due to excessive IFN-γ/IL-6 release.; and (4) the poor long-term persistence of CAR-T cells, resulting from T-cell exhaustion (as indicated by PD-1/TIM-3 upregulation) and host immune rejection, compromises long-term disease control. Together, these data underscore both the transformative potential and current limitations of CAR-T therapy in pediatric B-ALL.

The advent of CAR-T therapy represents a paradigm shift in the field of oncology, offering durable remissions for patients with previously untreatable hematological cancers. Nevertheless, this potent immunotherapy faces substantial barriers that limit its reach and impact (**Figure 2**). The most significant challenges are as follows: severe toxicities, such as CRS and ICANS; the persistent risk of relapse due to antigen escape or poor CAR-T cell persistence; and the formidable difficulty in achieving comparable success against solid tumors. Moreover, the presence of complex and costly manufacturing processes, significant delays in product delivery, and limited global accessibility creates substantial practical challenges. Addressing this intricate constellation of biological, clinical and socioeconomic difficulties is essential for advancing CAR-T therapy towards becoming a more widely applicable, safer, and sustainable treatment modality.

### 3.1 Toxicity of CAR T-cells

#### 3.1.1 Cytokine release syndrome

CRS is a systemic inflammatory response that is triggered by CAR-T cell activation. It is characterized by excessive cytokine production and immune hyperactivation (86). The underlying pathophysiology encompasses three interconnected phases: initial CAR-T cell activation, leading to IFN-γ and GM-CSF release (87–89); subsequent macrophage/monocyte activation, producing IL-6 and IL-1β (55, 90, 91); and ultimately endothelial dysfunction and organ injury, mediated by DAMPs (92, 93). and secondary hemophagocytic lymphohistiocytosis (sHLH) (56).

According to available research, the majority of patients with acute lymphoblastic leukemia (ALL) will experience CRS, with an incidence rate ranging from 77% to 100% (23, 56, 90). Furthermore, available evidence suggests that the incidence and severity of CRS are generally higher in patients with B-ALL receiving CD19-targeted CAR-T cell therapy compared to those with other B-cell malignancies such as non-Hodgkin lymphoma (94). This may be related to factors such as higher disease burden and a more inflammatory baseline state in advanced leukemia. Typically, patients develop initial symptoms within 14 days after receiving CAR-T cell therapy, with clinical manifestations exhibiting a distinct spectrum ranging from mild systemic symptoms commonly observed in Grade 1–2 cases (e.g., fever >38 °C and fatigue) to life-threatening manifestations, including refractory hypotension, acute respiratory distress syndrome (ARDS), and multi-organ failure, often accompanied by ICANS, manifesting as seizures or cerebral oedema (95). Current management strategies employ a risk-adapted approach (**Table 2**) guided by the 2019 consensus criteria established by the American Society for Transplantation and Cellular Therapy (ASTCT) (96). Recent in-depth studies of CRS have identified multiple predictive biomarkers that enable early intervention, including serum IL-6, IFN-γ, serum MCP-1, and others, all of which have a specificity of more than 90% for identifying patients at risk for severe progression of CRS (97).

**Table 2:** Current management strategies for CRS.

CRS grade	Symptoms	Intervention
1	Fever	Supportive care
2	Fever with hypotension responsive to fluids	Tocilizumab(8 mg/kg intravenous) ± supportive care
3	Hypotension requiring vasopressors	Tocilizumab + corticosteroids (e.g., dexamethasone 10mg q6h)
4	Hypotension, ARDS	Corticosteroids (98, 99)+organ support
Refractory	sHLH/DIC	Ruxolitinib (100)/anakinra (101)+ICU management

CRS, Cytokine Release Syndrome; sHLH, secondary Hemophagocytic Lymphohistiocytosis; DIC, Disseminated Intravascular Coagulation.

The evolving landscape of CRS research continues to provide valuable insights into its underlying biology and potential therapeutic targets. Single-cell sequencing technologies have revealed the pivotal function of the neutrophil-JAK/STAT axis (102, 103) in the initiation of CRS, wherein early neutrophil activation amplifies the inflammatory cascade, suggesting the possibility of “pre-storm” intervention strategies that preemptively dampen this initial trigger. Preclinical models show that engineering approaches to CAR-T cell design have the potential to mitigate excessive activation while maintaining therapeutic efficacy. These strategies include the incorporation of suicide switches (e.g., iCasp9) (104), which allow for the selective elimination of CAR-T cells via a small molecule activator, and the optimization of CAR structure (105) through fine-tuning of scFv affinity, hinge length, and co-stimulatory domains to achieve more controlled T-cell activation.

Looking ahead, the integration of dynamic biomarker profiling (e.g., serial monitoring of IFN-γ/IL-6 ratios and neutrophil activation markers) with artificial intelligence holds promise for creating predictive models that identify high-risk patients and enable personalized, preemptive management. These research advances underscore the critical importance of continued investigation into CRS pathophysiology and management. The prioritization of validation of predictive algorithms in multicenter trials, optimization of engineered CAR designs for clinical use, and development of standardized protocols for risk-adapted therapy should be the focus of future directions. Integrating artificial intelligence with real-time biomarker monitoring and advancing controllable CAR-T platforms are promising approaches to decoupling antitumor efficacy from inflammatory toxicity. As CAR-T therapy expands to new indications and patient populations, these innovations will be essential to ensuring the safety and effectiveness of this transformative treatment.

#### 3.1.2 Immune effector cell associated neurotoxicity syndrome

ICANS is a serious neurological complication of CAR-T cell therapy. It is driven by a cascade of events involving systemic inflammation, blood-brain barrier (BBB) disruption, and direct neurotoxicity (98). The pathogenesis is centered on cytokine-mediated endothelial activation (elevated Ang-2/VCAM-1), which leads to BBB breakdown (106, 107). This, in turn, permits immune cell infiltration into the CNS (108, 109). Once in the brain, activated microglia release reactive oxygen species and pro-inflammatory cytokines (110, 111). Downregulation of glutamate transporters causes excitotoxic neuronal damage through calcium overload (112) and synaptic dysfunction (113).

Clinically, ICANS presents with a variety of symptoms, including but not limited to language disorders(e.g., encephalopathy), cognitive impairments, and seizures. In severe cases, cerebral oedema may develop, with severity graded according to the ASTCT 2019 criteria from Grade 1 (mild word retrieval difficulty) to Grade 4 (coma or status epilepticus) (96). Typically, diagnostic evaluations reveal characteristic cerebrospinal fluid abnormalities, including elevated levels of IL-6 and CXCL10, as well as electroencephalogram (EEG) findings showing diffuse slow waves or epileptiform discharges (96), while they are primarily used in research settings rather than routine clinical practice due to limited availability and uncertain prognostic value. Given the high incidence of ICANS and its potential for rapid progression, prophylactic administration of antiepileptic agents is widely adopted in clinical practice. Levetiracetam is commonly used as standard seizure prophylaxis in patients receiving CAR-T cell therapy, particularly in those with high tumor burden or early neurological symptoms. This preventive strategy is typically initiated prior to or at the time of CAR-T cell infusion and continued during the period of highest ICANS risk (114). In established cases, current management strategies emphasize a multi-tiered approach that includes prevention, acute intervention and long-term neurorehabilitation (**Table 3**) (114). For ICANS that do not respond to corticosteroid therapy, alternative therapeutic interventions should be contemplated, encompassing anabolic acid, rucotinib, cetuximab, cyclophosphamide, antithymocyte globulin, or intrathecal hydrocortisone, with or without chemotherapy (115). Notably, the use of tocilizumab is controversial due to its limited ability to penetrate the BBB, though its use in refractory cases via intrathecal administration is being explored (83, 114, 116).

**Table 3:** Current management strategies for ICANS.

ICANS grade	Symptoms	Intervention
1	Mild word-finding difficulty	Supportive care (hydration, levetiracetam) + close monitoring
2	Difficulty with commands, disorientation	Corticosteroids + supportive care
3	Somnolence, seizures, focal signs	High-dose corticosteroids + ICU care
4	Coma, cerebral edema, status epilepticus	Aggressive immunosuppression + ICU management
Refractory	Steroid-resistant neurotoxicity	IL-1/JAK inhibition (anakinra/ruxolitinib) ± intrathecal therapy
Prophylaxis	—	Levetiracetam

ICANS, Immune Effector Cell Associated Neurotoxicity Syndrome.

Emerging strategies focus on prevention and treatment. They incorporate biomarker-based risk stratification (e.g., elevated baseline ANG2/ANG1 ratio (117)) to predict endothelial dysfunction, CAR-T cell engineering (e.g., GM-CSF/IL-6 knockdown and introduction of miR-146a to modulate cytokine release (118, 119)) to reduce the production of key cytokines that drive neuroinflammation, and novel neuroprotective approaches, such as glutamate transporter enhancers (120) to mitigate excitotoxicity and BBB-targeted nanoparticle delivery of anti-inflammatories (121) to improve CNS drug delivery. These advances aim to balance therapeutic efficacy with reduced neurotoxicity. However, optimal steroid regimens and reliable treatments for refractory ICANS require further validation through clinical studies. Future research will emphasize gaining mechanistic insights into excitotoxicity pathways and developing CNS-penetrant immunomodulators to improve outcomes in this potentially fatal complication.

#### 3.1.3 Other CAR-T related toxicities

In addition to CRS and ICANS, CAR-T cell therapy is associated with several other significant toxicities that require clinical recognition and management. Noteworthy examples include B-cell aplasia and hemophagocytic lymphohistiocytosis (HLH)/macrophage activation syndrome (MAS) (122).

B-cell aplasia is a predictable, on-target/off-tumor toxicity resulting from the destruction of normal, CD19-positive B cells, as well as malignant cells. This leads to prolonged hypogammaglobulinemia, which increases the risk of infection (123). Management consists of regular intravenous or subcutaneous immunoglobulin replacement therapy (IVIG/SCIG) to maintain protective antibody levels (115). The duration of B-cell aplasia correlates directly with CAR-T cell persistence and serves as a pharmacodynamic marker of functional activity, necessitating long-term monitoring in responders (90).

HLH/MAS is a severe, life-threatening hyperinflammatory syndrome that can overlap with or be triggered by severe CRS. It is characterized by sustained fever, cytopenias, hepatosplenomegaly, hyperferritinemia, coagulopathy, and hemophagocytosis (124). The pathophysiology involves uncontrolled activation of macrophages and lymphocytes, resulting in a cytokine storm often more intense than that seen in typical CRS (125). Management requires aggressive immunosuppression, often involving high-dose corticosteroids and the IL-1 receptor antagonist anakinra, in addition to standard CRS treatment. Etoposide may be considered for refractory cases following treatment guidelines for secondary HLH (126, 127).

Other reported toxicities include cardiovascular events, such as cardiomyopathy and arrhythmias, which may result from cytokine-mediated injury or hemodynamic instability due to CRS (128–131). Prolonged cytopenias following lymphodepleting chemotherapy and CAR-T infusion are also common and may require growth factor support or stem cell rescue in severe cases (132–135). Additionally, post–CAR-T infections, driven by prolonged cytopenias and B-cell aplasia, are common as well, with early bacterial and late viral or opportunistic patterns, highlighting the need for risk-adapted surveillance and prophylaxis (136–138).

### 3.2 Resistance mechanism of CAR-T cells

**Figure 3:**
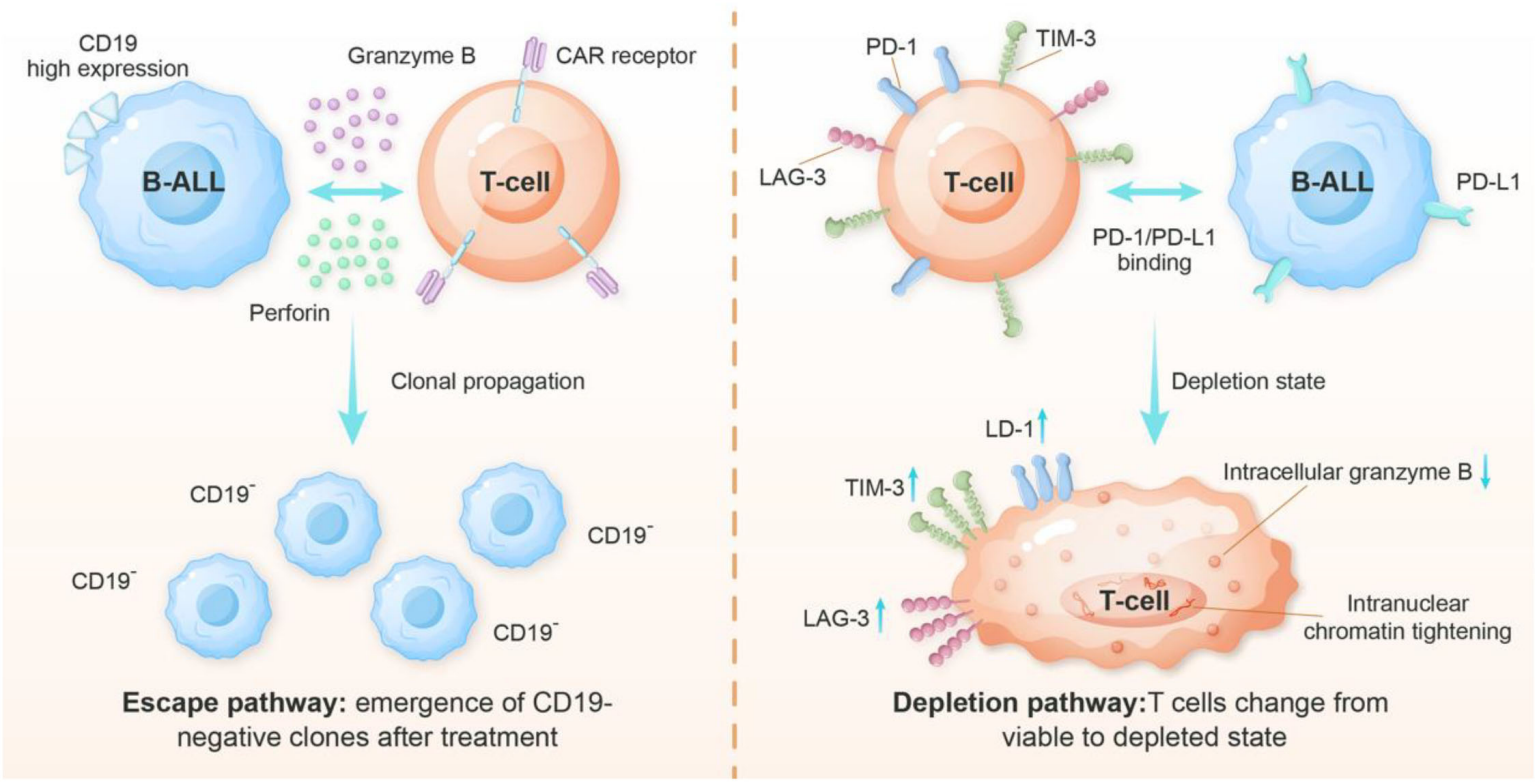
Mechanisms of antigen escape and T-cell depletion in CAR-T cell therapy. Antigen escape: Under therapeutic pressure, CD19-negative leukemic clones emerge and expand through clonal propagation, evading CAR-T cell recognition and leading to relapse. *For a detailed breakdown of the* sp*ecific molecular mechanisms (e.g., mutations, trogocytosis, lineage switch) that can initiate this process, please refer to***Table 4**.T-cell depletion: Exposure to persistent antigens drives CAR-T cells into an exhausted state. This state is characterized by the upregulation of inhibitory receptors (PD-1, LAG-3, and TIM-3) and the activation of the PD-1/PD-L1 pathway. This results in diminished cytotoxic function and reduced granzyme B, eventually leading to T-cell depletion.

The mechanisms underlying CAR-T cell resistance are complex and multifactorial, involving dynamic interactions between tumor cells, CAR-T cells, and the tumor microenvironment (TME). Primary resistance mechanisms include antigen escape through loss or downregulation of target antigens (e.g., CD19 in B-ALL or BCMA in multiple myeloma), intrinsic T-cell dysfunction (such as exhaustion or poor persistence), and immunosuppressive elements within the TME (including myeloid-derived suppressor cells and regulatory T cells). Additionally, emerging evidence suggests that metabolic competition and epigenetic modifications may further contribute to treatment failure. Understanding these resistance mechanisms is essential for developing next-generation CAR-T cell therapies with improved efficacy and durability (**Figure 3**).

#### 3.2.1 Antigen escape

Antigen escape is a significant challenge that limits the long-term efficacy of CAR-T cell therapy for B-ALL. It often manifests as aggressive disease relapses that are resistant to subsequent CD19-targeted therapies and has particularly severe clinical consequences. This occurs in 20%-30% of relapsed cases following CD19-targeted immunotherapy. Some studies have shown that CD19-negative relapse rates exceed 30% in certain high-risk subgroups (139). This resistance mechanism develops when leukemic blasts evade immune surveillance through various alterations in the expression of target antigens (**Table 4**). The most common form involves complete loss of CD19 surface expression, which can result from either selection of pre-existing CD19-negative clones or acquired genetic modifications under therapeutic pressure (140, 141). Notably, specific CD19 mutations, particularly exon 2 deletions (Δex2), produce truncated protein variants that retain intracellular domains but lack the membrane-anchoring region critical for CAR-T recognition. Additionally, alternative splicing events can generate CD19 isoforms with modified extracellular epitopes, while epigenetic silencing through promoter hypermethylation represents another pathway to antigen downregulation (142–144).

**Table 4:** Mechanistic differences in CD19 antigen escape.

Mechanism type	Key alteration	Molecular biasis	Clinical impact
Alternative splicing (142)	Δex2 deletion	Produces non-functional CD19	CD19 mRNA present but surface protein absent.
Genetic mutations (145)	CD19 gene mutations	Disrupt CD19 protein production	Complete antigen loss
Inmmune selection (146)	Pre-existing CD19-negative clones expand	Pre-existing CD19-negative clones become dominant.	Higher relapse risk
Epitope masking (147)	Leukemic cells express CAR transgene	CAR binds CD19 in cis, masking it from detection and immune attack.	False-negative flow cytometry results
Lineage Switch (148)	B-cell → myeloid transformation	Loss of B-cell genes (PAX5, EBF1); gain of myeloid genes (CEBPα).	Poor prognosis, common in KMT2A-rearranged B-ALL.
Low Antigen Density (149)	Downregulation of CD19 surface expression	Promoter hypermethylation or reversible CAR-T-induced suppression.	Diminished CAR-T efficacy
Trogocytosis (150)	CD19 transfer to CAR-T cells	CAR-T cells “nibble” CD19 from leukemic cells, reducing target density.	CAR-T fratricide and antigen escape.

#### 3.2.2 CAR-T cell exhaustion and persistence

Another cause of relapse in CAR-T cell therapy is the short-lived presence of CAR-T cells, which manifests as CD19-positive relapses (139). A retrospective study found that patients experiencing CD19-positive relapses lost tisagenlecleucel persistence more rapidly than those who achieved durable remissions (151).Clinical trials have demonstrated that the longevity of CAR-T cells can differ greatly, with CAR-Ts that utilize the 4-1BB co-stimulatory domain exhibiting a median persistence that is notably longer than CAR-Ts that use the CD28 co-stimulatory domain (152–154). The choice of co-stimulatory domain may play a fundamental role; CD28-based CARs typically induce strong initial expansion, but lead to rapid contraction due to activation-induced cell death. In contrast, 4-1BB-based constructs promote longer persistence through enhanced mitochondrial biogenesis and oxidative metabolism (155, 156).

CAR-T cell exhaustion represents a progressive functional deterioration characterized by hierarchical loss of effector functions. This complex biological process initiates with chronic antigen stimulation, which triggers sustained TCR/CD3ζ signaling through the CAR construct (157). The resulting persistent activation leads to upregulation of multiple inhibitory receptors, including PD-1, TIM-3, LAG-3, and CTLA-4, which collectively establish an immune checkpoint barrier (158). At the molecular level, exhaustion is mediated by dynamic epigenetic reprogramming involving the coordinated action of transcription factors such as NR4A family members (NR4A1, NR4A2, NR4A3) and TOX (159–161). These factors induce chromatin remodeling that stabilizes the exhausted phenotype, creating an epigenetic “lock” that is increasingly difficult to reverse as exhaustion progresses. The functional consequences of exhaustion manifest in a stepwise manner. Early-stage exhausted CAR-T cells initially retain proliferative capacity and cytokine production (particularly IL-2), while late-stage exhaustion is marked by complete loss of proliferative potential and severely impaired cytokine secretion (IFN-γ, TNF-α) (162). This functional decline correlates with metabolic alterations, including reduced glycolytic flux and oxidative phosphorylation, as well as mitochondrial dysfunction characterized by decreased membrane potential and increased reactive oxygen species (ROS) production (163). Recent single-cell RNA sequencing studies have identified distinct exhaustion subpopulations with varying degrees of dysfunction, suggesting the existence of potentially reversible intermediate states (164, 165).

#### 3.2.3 Tumor microenvironment suppression

Furthermore, real-world data indicated that CAR-T persistence was superior in low-tumor-load (<50% bone marrow primordial cells) and younger patients (<18 years old), with a 24-month RFS rate of 50.3%. In contrast, patients with a high-tumor load or those who had been treated with inotuzumab had a more unfavorable prognosis (166). The immunosuppressive tumor microenvironment further impairs CAR-T cell persistence through multiple mechanisms, illustrating its impact on CAR-T cell therapy. Myeloid-derived suppressor cells (MDSCs) and regulatory T cells (Tregs) create an inhibitory niche through secretion of IL-10, TGF-β, and arginase-1, while physical barriers like dense extracellular matrix components impede CAR-T cell trafficking and function (167, 168). Metabolic competition is particularly intense in solid tumors, where cancer cells actively deplete glucose and tryptophan while producing lactate, creating an energetically hostile environment for CAR-T cells (163, 169).

In summary, the main resistance mechanisms of CAR-T cell therapy for B-ALL include antigen escape, T cell depletion, and an immunosuppressive microenvironment. The development of targeted strategies to counter each of these barriers—such as dual-targeting CARs for antigen escape, checkpoint inhibition for T-cell exhaustion, and microenvironment modulation—forms a critical focus of ongoing research. Future studies should focus on optimizing these strategies’ implementation and establishing a reliable efficacy prediction system.

## 4 Novel strategies for CAR-T therapy

**Figure 4:**
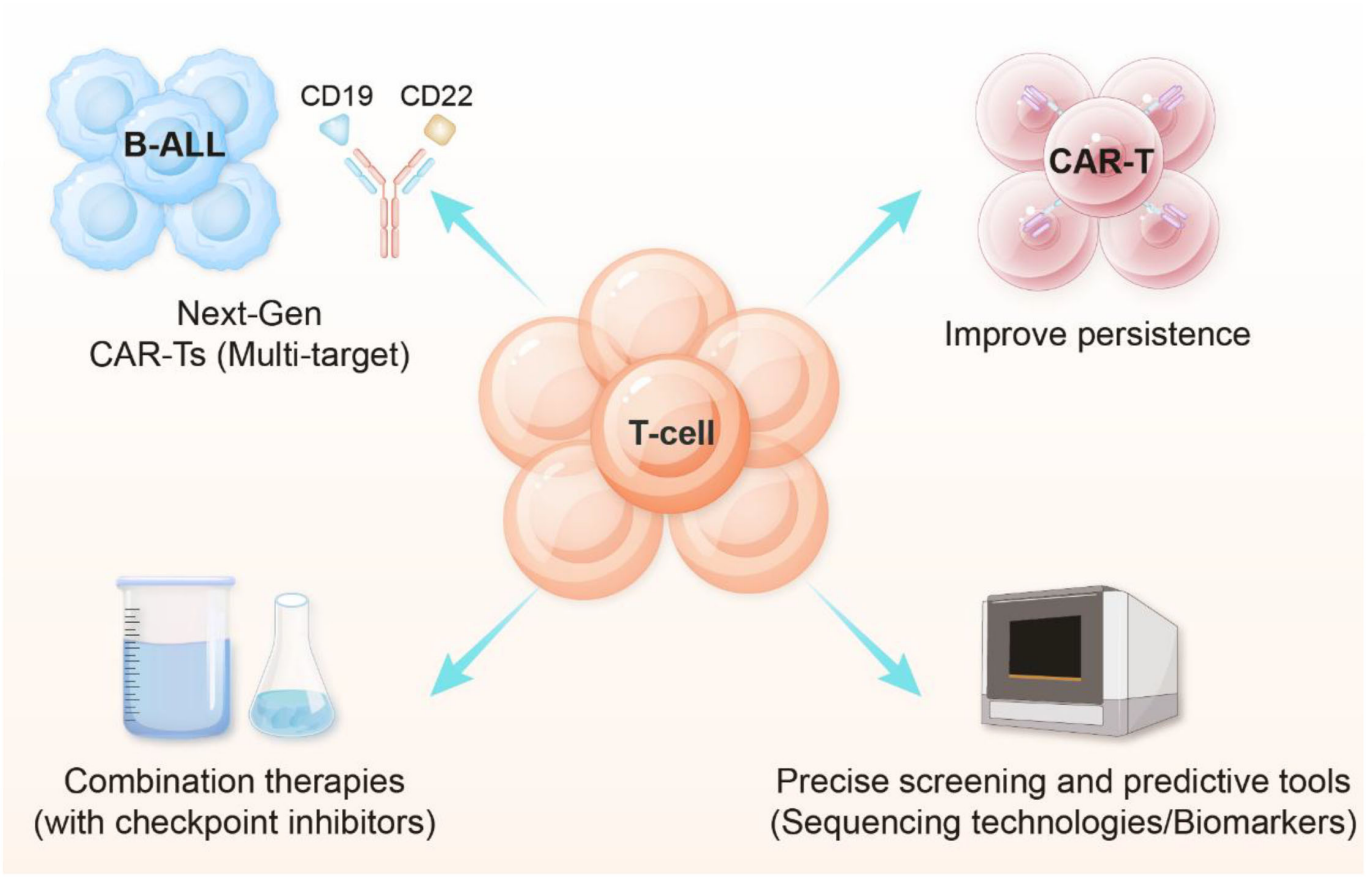
Key strategies for optimizing CAR-T cell therapy in B-ALL.

Building upon the resistance mechanisms discussed in the previous section, current research has focused on developing innovative strategies to enhance the efficacy and safety of CAR-T therapy. As illustrated in the **Figure 4**, the key approaches encompass several cutting-edge directions, including: (1) next-generation CAR-T designs, particularly multi-targeting constructs (e.g., CD19/CD22) to mitigate antigen escape; (2) T-cell engineering to improve persistence and functionality; (3) rational combination therapies with immune checkpoint inhibitors or other immunomodulators; and (4) advanced screening tools leveraging sequencing technologies and predictive biomarkers for patient stratification. Collectively, these strategies—which include improved detection, enhanced therapeutic efficacy, and optimized treatment integration—form a comprehensive framework to overcome current limitations in CAR-T therapy. Ultimately, the aim is to achieve more durable responses and better outcomes for pediatric B-ALL patients.

### 4.1 Muti-targeted CAR-T in B-ALL

Antigen escape remains one of the main causes of treatment failure. To address this issue, multi-targeted CAR-T cell therapies have emerged. Among these, CD19/CD22 dual-targeted CAR-T cells have shown considerable promise. Dual-targeted CAR-T works by simultaneously recognizing both CD19 and CD22 antigens. Even if one antigen is downregulated or lost, the CAR-T can maintain anti-tumor activity through the other target, allowing dual-targeted CAR-T to continue to be effective (170). A retrospective study found that CD19/CD22 dual-targeted CAR-T therapy resulted in CR rates of up to 98% in pediatric and adult B-ALL patients. This rate was significantly higher than the 83% rate for single-targeted CAR-T therapy. Furthermore, patients who received tandem CD19/22 CAR-T therapy followed by hematopoietic stem cell transplantation had higher CR rates (28.5% vs. 70.5%) (171). Additionally, novel logic-gated CAR-Ts (e.g., AND-gate or OR-gate designs) improve target specificity and reduce off-target toxicity. The GD2/B7-H3 AND-gate CAR-T, for instance, activates only in the presence of both antigens, thereby enhancing selective tumor killing (172). Furthermore, triple-targeting CAR-T constructs (e.g., simultaneous targeting of CD19, CD22, and CD20) are currently under investigation in early-phase clinical trials (173, 174). This strategy aims to further broaden antigen coverage and minimize the risk of escape due to the loss or downregulation of any single antigen. However, multi-targeted CAR-Ts face challenges including increased manufacturing complexity, a potentially elevated risk of CRS, and insufficient CAR-T durability. The AMELIA trial showed that, despite an initial CR rate of 86% for dual-targeted CAR-T (AUTO3), the one-year event-free survival rate was only 32% (175). This suggests that CAR-T durability needs optimization. Overall, multi-targeted CAR-T therapy is a significant advancement in the treatment of B-ALL, but further optimization is necessary to enhance long-term efficacy and minimize toxicity.

### 4.2 Armored CAR-Ts

Armored CAR-T cells (Armored CARs) represent a breakthrough in B-ALL therapy. These novel CAR-T cells are genetically engineered to secrete immunomodulatory factors or express co-stimulatory molecules, significantly enhancing their anti-tumor activity and durability (176). In terms of cytokine-enhanced CAR-T, IL-12-secreting CAR-T cells can directly kill tumor cells and activate the immune system. Preclinical studies have shown that IL-12-secreting CAR-T cells significantly improve the tumor clearance rate in a B-ALL mouse model (177), meanwhile, IL-15-enhanced CAR-T cells promote T-cell survival and memory phenotyping (81). Preclinical studies showed that targeting the immunosuppressive properties of the tumor microenvironment with a PD-1/CD28-converting receptor design can overcome PD-L1-mediated inhibition (178). Additionally, TIM-3 decoy CAR-T cells were shown to prevent the formation of memory phenotypes by blocking the Galectin-9/TIM-3 immunosuppressive pathway (179). This demonstrates their potential to promote prolonged survival in preclinical models. Despite these significant efficacy gains, challenges remain, such as managing toxicity and optimizing the preparation process. Future directions include developing general-purpose armored CAR-T products.

### 4.3 Sequential or combination therapies

Researchers are focusing on sequential or combination therapy strategies to improve long-term prognosis. Available clinical study data suggest that these strategies can significantly improve long-term prognosis. A phase II clinical trial from the U.S. National Cancer Institute (NCT03518112) showed that, among B-ALL patients who were MRD-positive after CD19 CAR-T therapy, 78% of patients who received consolidation therapy with Blinatumomab became MRD-negative, with an 18-month disease-free survival rate of 68% (180). For patients with high-risk factors, including high disease burden at the time of CAR-T infusion (181), early loss of CAR-T cell persistence, and the presence of very high-risk genetic abnormalities, such as TP53 mutations (182), KMT2A rearrangements, or complex karyotypes (183), etc., CAR-T cell therapy followed by allogeneic hematopoietic stem cell transplantation (allo-HSCT) can improve long-term survival, although some aspects remain under investigation. This is an important option for consolidating the outcomes of patients with B-ALL, but there is a lack of clinical studies designed specifically to evaluate the role of allo-HSCT after CAR-T cell therapy. In addition, the combination of CAR-T and epigenetic modulators has demonstrated synergistic effects. A study examined the use of decitabine (100 mg/m² total dose administered over three days) in conjunction with fludarabine/cyclophosphamide (FC) as a lymphodepleting regimen, followed by CD19/CD22 dual-targeted CAR-T therapy. While there was no significant difference in complete remission rates between groups 28 days after CAR-T infusion, the decitabine group showed a significant long-term survival advantage, with 3-year overall survival and leukemia-free survival rates of 92.3% and 92.9%, respectively, compared to 41.7% and 27.3% in the control group (P = 0.005 and P < 0.001, respectively) (184). Mechanistic studies have demonstrated that decitabine enhances the efficacy of CAR T-cell therapy by increasing the expression of tumor antigens, such as CD19 and CD22. Furthermore, decitabine has been shown to have a favorable safety profile, with all adverse events being reversible and manageable (185).In patients with relapsed or refractory B-ALL, CAR-T therapy combined with a PD-1 inhibitor showed enhanced anti-tumor activity and more durable remissions. A multicenter, phase II trial (NCT02650999) evaluating the combination of pembrolizumab and CD19 CAR-T therapy showed that the six-month sustained remission rate was 63% in the combination group, which is significantly higher than the 33% rate in the CAR-T alone group (186). This difference may be due to the reversal of T-cell depletion and remodeling of the tumor microenvironment. However, the safety of these combination strategies, particularly the management of immune-related adverse reactions, still needs to be carefully evaluated.

### 4.4 Next-gen CAR designs

Next-generation CAR-T designs are making B-ALL treatments safer and more controllable. Universal CAR-T (UCAR-T) significantly reduces the risk of GVHD by knocking down the HLA molecule and TCR of T cells using CRISPR-Cas9 gene editing technology. Several UCAR-T products have entered clinical trials, and preparation time can be shortened to two to three weeks, greatly improving treatment accessibility (187). Modulable CAR-T systems enable precise modulation of CAR-T activity by introducing small molecular switches or photocontrol elements. These systems typically rely on small molecule-dependent dimerization domains, such as the rapamycin-based iMC system, where administration of the small molecule drug induces CAR dimerization and activation. For example, CAR-T systems that use rapamycin or its analogues as molecular switches can be rapidly inactivated in the event of severe toxicity (188). However, the effectiveness of molecular safety switches in enhancing safety while reducing toxicity in CAR-T cell-based therapies remains to be seen, as they have not yet undergone clinical testing. Hypoxia-inducible CAR-Ts are activated by hypoxic conditions specific to the tumor microenvironment, which have demonstrated effective tumor targeting and safety in preclinical studies (189). Additionally, transient expression CAR-T, based on mRNA technology, provides a new solution for treating acute toxic reactions with a controlled duration of CAR expression, significantly improving safety, but requires repeated administration for sustained efficacy (190). These innovative designs address the limitations of traditional CAR-T and open new avenues for precision therapy. Due to their compatibility with existing infrastructure, demonstrated safety, and real-time activity control, modulable CAR-T systems currently offer the greatest near-term clinical potential. UCAR-T shows promise for long-term use, pending improvements in persistence, while hypoxia-inducible designs await better biomarkers. A tiered approach combining these technologies may optimize outcomes. Future priorities should include head-to-head trials and cost-effectiveness analyses to guide clinical implementation and ensure equitable access.

## 5 Conclusion

In all, CD19-targeted CAR T-cell therapy has emerged as a breakthrough treatment for R/R B-ALL, offering promising remission rates (23, 24, 56, 69, 191). CAR-T cell therapy has effectively transformed the treatment paradigm for pediatric B-ALL, particularly in cases that are relapsed or refractory. The clinical outcomes have demonstrated unparalleled complete remission rates, providing novel therapeutic hope where conventional approaches have historically fallen short. The success of CD19-targeted CAR-T products has established cellular immunotherapy as a cornerstone of modern B-ALL management.

Nevertheless, a number of challenges must be addressed if this therapy is to realize its full potential. Treatment-related toxicities and resistance mechanisms continue to limit clinical outcomes, while prolonged manufacturing times and high associated costs significantly restrict patient accessibility and global scalability. The individualized manufacturing process, requirement for specialized centers, prolonged production timelines, and intensive supportive care contribute to substantial treatment costs. These constraints disproportionately limit access in resource-limited settings and may delay therapy in patients with rapidly progressive disease. Although emerging strategies such as automated manufacturing platforms, point-of-care production, and allogeneic “off-the-shelf” CAR-T products hold promise for reducing costs and improving availability, their clinical scalability and long-term cost-effectiveness require further validation. These limitations have catalyzed the development of next-generation solutions, including advanced engineering approaches and rational combination strategies.

To ensure future progress, innovative solutions are imperative, and these solutions must be implemented across multiple fronts. The application of artificial intelligence-driven multi-omics profiling holds promise for enabling precise toxicity prediction and patient selection. Interventions that target the microenvironment, such as TGF-β blockade (192) or IDO1 inhibition (193), have the potential to enhance the persistence of CAR-T cells. The implementation of automated closed-system manufacturing and point-of-care production platforms has the potential to enhance scalability and reduce costs.

As the field progresses, it will become imperative to integrate state-of-the-art technologies with foundational immunological insights. By addressing current limitations through multidisciplinary innovation, CAR-T therapy has the potential to evolve into a more precise, durable, and globally accessible treatment, ultimately improving outcomes for all pediatric B-ALL patients.
