# Supplementary material for: Precision immunotherapy with CAR-T cells in pediatric B-cell acute lymphoblastic leukemia: advances and unanswered challenges
Source: Front Oncol. 2026 Jan 14;15:1691189. doi: 10.3389/fonc.2025.1691189 (PMC12846953; doi:10.3389/fonc.2025.1691189)
Supplement: Supplementary file 1 [file SupplementaryFile1.docx]

**Table S1:** Trials summarized strictly from the 165 references provided by the author. Columns reflect the reviewer’s request: trial number, target, research institution, and CAR-T type. For studies where the trial number/registry is not explicitly listed in the cited reference, a slash “/” is used.

| Trial / Study (Ref IDs) | Trial Number / Registry | Target(s) | Research Institution / Sponsor | CAR-T Type (if available) |
| --- | --- | --- | --- | --- |
| ELIANA: Tisagenlecleucel in pediatric/YA R/R B-ALL ([17], [59]) | NCT02435849 | CD19 | Multicenter; CHOP/UPenn lead; Sponsor: Novartis | Tisagenlecleucel (4-1BB) |
| MSK adult CD19 CAR-T (19-28z) long-term follow-up ([133]) | / | CD19 | Memorial Sloan Kettering (MSK) | 19-28z (CD28 costimulation) |
| ZUMA-3: Brexucabtagene autoleucel in adult R/R B-ALL ([134]) | NCT02614066 | CD19 | Multicenter; Sponsor: Kite Pharma | Brexucabtagene autoleucel (CD28) |
| NCI CD22 CAR-T dose-escalation (mixed age) ([72]) | NCT02315612 | CD22 | National Cancer Institute (NCI) | CD22 CAR-T (4-1BB) |
| Defined-composition pediatric/YA CD19 CAR-T ([18]) | / | CD19 | Seattle Children’s / Fred Hutch (per author list) | CD19 CAR-T (defined composition) |
| Dual-target CD19/CD22 CAR-T — adults (phase 1, mixed B malignancies; B-ALL subset) ([73]) | / | CD19 + CD22 | Multicenter (per publication) | Dual-target CAR-T |
| Dual-target CD19/CD22 CAR-T — pediatric/YA (phase 1) ([153]) | / | CD19 + CD22 | Great Ormond Street Hospital / UCL (per publication) | Dual-target CAR-T（low-affinity CD19 binder reported） |
| Bicistronic CD19/22 CAR-T — pediatric/YA (phase 1) ([82]) | / | CD19 + CD22 | NCI | Bicistronic CD19/22 CAR-T |
| Sequential CD19→CD22 CAR-T — children (Blood 2020) ([79]) | / | CD19 then CD22 (sequential) | China（per publication） | Sequential autologous CAR-T (CD19 then CD22) |
| Sequential CD19→CD22 CAR-T — 5-year outcomes (Haematologica 2025) ([74]) | / | CD19 then CD22 (sequential) | China（per publication） | Sequential autologous CAR-T (CD19 then CD22) |
| CRISPR/Cas9-engineered universal dual-target CD19/CD22 CAR-T (Clin Cancer Res 2021) ([23]) | / | CD19 + CD22 | China（per publication） | Allogeneic CRISPR/Cas9-engineered dual-target CAR-T |
| Decitabine-primed lymphodepletion → bispecific CD19/CD22 CAR-T (Exp Hematol Oncol 2023) ([159]) | / | CD19 + CD22 | China（per publication） | Bispecific CD19/CD22 CAR-T with DAC + F/C priming |
| Early CHOP/UPenn pediatric CD19 CAR-T (first-in-pediatric reports) ([58], [87]) | / | CD19 | CHOP / UPenn | CTL019 / CD19 CAR-T (4-1BB) |

“/” = the trial number/registry is not explicitly stated in the corresponding cited reference; kept blank intentionally to remain within the provided sources only.
